# Evolution of Media Supporting the Development of Mammalian Preimplantation Embryos In Vitro

**DOI:** 10.3390/biology13100789

**Published:** 2024-10-01

**Authors:** Hongsheng Men

**Affiliations:** 1 Mutant Mouse Resource and Research Center, Columbia, MO 65201, USA; menho@missouri.edu; 2 Rat Resource and Research Center, Columbia, MO 65201, USA; 3 Department of Veterinary Pathobiology, University of Missouri-Columbia, 4011 Discovery Drive, Columbia, MO 65201, USA

**Keywords:** chemically defined media, embryo culture, embryo development, development arrest

## Abstract

**Simple Summary:**

The media supporting selected mammalian preimplantation embryos in vitro have played an essential role in the advancement of assisted reproductive technology (ART). ART has in turn transformed biomedical research with genetically modified, disease-specific animal models, agriculture with genetically modified farm animals and human reproductive health with infertility treatment. However, the current culture systems still cannot replicate the in vivo environment, and embryos derived from in vitro systems are poor in quality compared to their in vivo counterparts. The objective of this review is to discuss the lineage of chemically defined media from simple salt solutions to modern media commonly used in embryo cultures for laboratory animals (mice and rats) and farm animals (pigs and cattle) and provide some insights for future improvements. This discussion provides readers with knowledge regarding the evolution of media used to support the development of preimplantation embryos in vitro and aims to inspire interest in the development of media to better support embryo development in vitro.

**Abstract:**

Assisted reproductive technology has revolutionized our ability to genetically manipulate, maintain and rederive laboratory animals of biomedical importance; manipulate animal reproduction or genetics to boost production of farm animals; and improve human reproductive health. The media for in vitro manipulation and the culture of embryos play a critical role in the development of assisted reproductive technology. In this review, the evolution of culture media supporting embryo development in vitro from selected animal species, laboratory animals (mice and rats) and farm animals (pigs and cattle), will be discussed with a focus on the development of chemically defined media.

## 1. Introduction

Technology development in the manipulation of mammalian gametes in vitro has enabled us to create, maintain and rederive mutant animals of biomedical importance; manipulate animal reproduction to boost production of farm animals with superior genetic merit; genetically modify farm animals to produce animals with altered traits such as disease resistance, wool growth, body growth and milk composition; and treat human infertility. Media supporting the development of preimplantation embryos in vitro play a critical role in the advancement and application of assisted reproductive technology in various species. Excellent reviews exist describing the culture and medium development of preimplantation embryos in individual species, such as mouse [1,2,3], rat [4], swine [5] and bovine [6,7]. Due to the competitive nature for media used in human embryo culture, it is difficult to delineate the ingredients of human embryo culture media currently in use. Therefore, we will focus on the evolution and lineage of modern chemically defined culture media used for laboratory animal (mice and rats) and farm animal (swine and bovine) embryo cultures. As most modern media can be traced back to Ringer’s solution, we will start with a brief introduction of Ringer’s solution and its selected derivatives. Then, for individual species, we will start with a discussion of the most recent media and then trace back to its precursor media.

**Figure 1:**
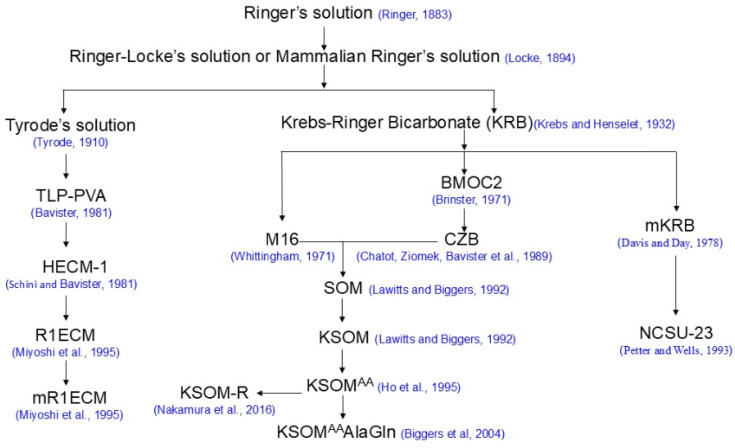
Selected mammalian embryo culture media with a Ringer’s solution origin [8,15,16,19,20,22,23,27,29,37,38,41,43,44,47].

Historically, there are different strategies for formulating embryo culture media: the so-called “let the embryo choose” approach and the “back to nature” approach [2]. Most of the modern media used for mammalian embryo cultures fall into the “let the embryo choose” approach and can be traced back to Ringer’s solution (Figure 1), which was formulated by Sydney Ringer [8]. The best examples of the “back to nature” approach are synthetic oviductal fluids (SOFs), human tubal fluid and porcine zygote mediums (PZMs), which were formulated based on the biochemical composition of sheep, and human and porcine fallopian tubal fluids, respectively [9,10,11].

## 2. Ringer’s Solution and Its Selected Derivatives

Ringer’s solution was developed by Sydney Ringer (1835–1910), a British clinician, physiologist and pharmacologist, based on his research identifying components that were able to replace blood to sustain normal cardiac function ex vivo using isolated heart tissues from a frog (*Rana Temporaria*) [12,13,14]. There are various versions of Ringer’s solution, and the one in Table 1 is derived from its original publication [8,13]. Ringer studied the effects of electrolytes on cardiac and involuntary muscles and, in particular, the actions of various inorganic salts on the behavior of the heart. In 1882, he developed a solution of salts dissolved in water to create an isotonic solution relative to animal body fluids. A selected list of solutions derived from Ringer’s solution which are related to the development of modern media used today is shown (Table 1). Ringer–Locke’s solution, also known as mammalian Ringer’s solution, differs in that it contains glucose and a higher amount of sodium chloride than the original solution [15]. Krebs–Ringer Bicarbonate (KRB) was modified from Ringer–Locke’s solution by Hans Krebs with increased glucose and sodium bicarbonate [15]. Tyrode’s solution was also altered from Ringer–Locke’s solution by Maurice Vejux Tyrode to contain magnesium, bicarbonate and large proportions of phosphoric acid and is often gassed with carbon dioxide. Like Ringer–Locke’s solution, Tyrode’s solution also contains glucose [15].

## 3. Mouse Embryo Culture Media

Potassium simplex optimized medium (KSOM, Table 2) is currently the medium used for mouse preimplantation embryo culture [16,17]. Compared to previous media, this medium is very versatile and supports mouse embryo development in vitro from the one-cell stage to blastocysts of various strains (outbred and inbred). The development of KSOM was driven by the effort to develop a medium that could overcome development arrest, a phenomenon where mouse embryos failed to proceed beyond the two-cell stage when cultured in vitro [18]. In order to develop a medium that could support mouse embryos from zygotes to blastocysts, an approach called simplex optimization was used to optimize the concentrations of eight components (NaCl, KCl, KH_2_PO_4_, MgSO_4_, sodium lactate, pyruvate, glucose and BSA) from existing M16 and two components (glutamine and EDTA) from existing Chatot–Ziomek–Bavister (CZB) media [16,19,20,21]. This effort led to the formulation of the simplex optimized medium (SOM) [22], which overcame the two-cell block. The concentrations of NaCl and KCl were later increased based on the intracellular Na^+^ and K^+^ concentrations in blastomeres of the two-cell mouse embryos, and this medium was named potassium simplex optimized medium (KSOM) [17]. Ho et al. (1995) demonstrated that the supplementation of half-strength (0.5×) Eagle’s minimal essential medium (MEM) essential amino acids (EAAs) and non-essential amino acids (NEAAs) in KSOM promoted mouse embryo development, and there are marked increases in cell numbers in expanded blastocysts compared to those cultured in KSOM only [23]. Therefore, half-strength (0.5×) Eagle’s MEM essential and MEM non-essential amino acids became the standard supplement of KSOM and was named KSOMaa [23]. Glutamine is an essential amino acid for preimplantation embryo development [2,22,24]. However, the spontaneous breakdown of glutamine due to its inherent instability and the by-products of glutamine metabolism are toxic to cultured embryos [25]. As a result, the more stable dipeptide form of glutamine, alanyl-L-glutamine, has been used to replace glutamine in the culture of mouse preimplantation embryos [26]. Though another dipeptide form of glutamine, glycyl-L-glutamine, has been demonstrated to increase the numbers of inner cell mass and trophoblasts [27], alanyl-L-glutamine is still commonly used since it is commercially available (e.g., GlutaMax by Thermo Fisher Scientific, Waltham, MA, USA).

One of the base media for SOM, the M16 medium, was developed in 1971 by modifying Kreb–Ringer bicarbonate (KRB) containing a portion (23 mM) of sodium chloride with isomolar (23 mM) sodium lactate to reduce the Cl^−^ concentration [19]. The second base medium of SOM, CZB medium, was developed in 1989 by excluding glucose and supplementing 1 mM glutamine in a modified Brinster’s medium for ovum culture (BMOC)-2 to overcome the two-cell block in mouse embryos cultured in vitro [20,28,29]. BMOC was developed by the supplementation of sodium lactate to KRB, which supports the development of mouse embryos in vitro from the two-cell stage [30].

## 4. Rat Embryo Culture Media

There are two media (Table 3) in use for rat preimplantation embryo culture from zygotes to blastocysts: the modified rat one-cell stage embryo culture medium (mR1ECM) and the KSOM for rat (KSOM-R) [31,32]. Rat embryos have also shown developmental arrest in culture at the two-cell stage [33,34]. Therefore, hamster embryo culture medium 1 (HECM 1) [31], which can support hamster embryo development from zygotes to blastocysts in vitro, has been used to culture rat embryos [35]. When first cultured in HECM 1, a limited number of rat embryos developed from zygotes into the blastocyst stage. Later, it was found that an osmolality lower than the iso-osmolality (290 mOsm) promotes rat embryo development in vitro [36]. Therefore, HECM 1 was modified by reducing the concentration of NaCl from 98.0 mM to 78.8 mM, eliminating amino acids and supplementing 7.5 mM glucose. This medium was named rat one-cell embryo culture medium (R1ECM) [37]. R1ECM was further modified by supplementation of Eagle’s EAA at 2% (*v*/*v*) and NEAA at 1% (*v*/*v*) and was then named modified R1ECM (mR1ECM) [38]. When mR1ECM was used for rat IVF, it was demonstrated that BSA and a higher osmolality (310 mOsm) increased by NaCl are beneficial for sperm penetration and pronuclear formation. This modification was named mR1ECM-BSA [39]. The supplementation of fetal bovine serum (FBS) to mR1ECM (mR1ECM-FBS) when rat embryos were at the early morula stage promoted the development of morula to blastocysts [40].

The development of HECM1 was achieved through a two-step modification of Tyrode’s solution. Tyrode’s solution was first modified by the supplementation of 0.5 mM sodium pyruvate and 1 mg/mL polyvinylalcohol (PVA) and was denoted as TLP-PVA [41]. In an effort to overcome the hamster embryo developmental block at the two- to four-cell stage, TLP-PVA was further modified by eliminating phosphate and glucose, which were identified to be the cause for two-cell block in cultured hamster embryos [42]. This medium was additionally supplemented with amino acids that can support hamster embryos from zygotes to the 8-cell stage without developmental arrest at the 2–4 cell stages. This medium was then termed HECM 1 [42].

Though mR1ECM can support rat embryos from zygotes to blastocysts in vitro and embryos resulting from culture in mR1ECM can develop to term after transferring into pseudopregnant recipients [38], mR1ECM is not optimal for rat embryo development in vitro as the post-implantation rate remains low. Therefore, as a high-performance medium, KSOM was optimized for rat embryo culture. After analysis of the amino acid profile in rat oviductal fluid, it was found that taurine, glycine, glutamate and alanine are rich in rat oviductal fluid [32]. Therefore, these four amino acids are supplemented into KSOM at 1 mM each. Additional modifications are the elimination of phosphate and supplementation of MEM EAA and MEM NEAA at their full strength (1×) [32]. It has been demonstrated that KSOM-R is more optimal for rat embryo development in vitro than mR1ECM [43].

## 5. Porcine Embryo Culture Media

Historically, there are a few media that have been used for porcine embryo culture [44]. However, a medium that successfully supported the entire preimplantation development of porcine embryos in vitro was not developed until the early 1990s. North Carolina State University-23 (NCSU-23) is the first medium that can support porcine embryos from zygotes to blastocysts with relatively high efficiency [45]. More recently, another medium, called the porcine zygote medium (PZM), has been shown to be more optimal for porcine embryo development in vitro [10].

NCSU-23 was developed by modifying mKRB with supplementation of glutamine, taurine and hypotaurine [45,46]. The supplementation of glutamine enables porcine embryos to overcome the developmental block, which occurs at the four-cell stage. The mKRB used for the optimization of porcine embryo culture was modified from KRB by supplementing glutamine, BSA, penicillin and streptomycin [47].

As mentioned above, PZM was developed by the “back to nature” strategy. It was formulated based on the published concentrations of inorganic compounds and energy substrates of pig oviductal fluid [10,48,49]. There are two versions of PZM: PZM-3 with BSA supplementation and PZM-4, a chemically defined version of PZM, with polyvinyl alcohol (PVA) supplementation in place of BSA. Both PZM-3 and PZM-4 support porcine embryo preimplantation development from zygotes to blastocysts. There are more cells in the inner cell mass (ICM) and a greater total cell number in Day 6 embryos derived from PZM-3 or PZM-4 compared to what is observed in embryos cultured in NCSU-23. Though the quality of PZM-4-generated embryos is lower than their in vivo-derived counterpart, the farrow rates after transfer are similar to those derived in vivo [10]. The compositions of those media are shown in Table 4.

## 6. Bovine Embryo Culture Media

Similar to human embryo culture media, bovine embryo culture media are currently also available commercially. Due to the proprietary nature of these commercial media, the exact ingredients are generally not disclosed. Therefore, the discussion of bovine embryo culture media will be focused on the published papers. There are diversified media, such as SOF [9,50], bovine embryo culture medium (BECM) [51], CR1aa [52] and KSOM [53,54,55], that are able to support bovine embryo development in vitro. Among these media, SOF has gained popularity in recent years [6,7,56,57]. SOF was originally formulated based on the chemical components of sheep oviductal fluid [9]. The original medium does not support optimal development of bovine one-cell embryos in vitro as most of them stalled after 6 days of culture [9]. Therefore, several modifications to SOF have been made to optimize its ability to support bovine embryo development in vitro (Table 5). The first optimization is the supplementation of Eagle’s MEM EAA and NEAA amino acids to SOF, and this medium was termed as SOFaa [58]. Though it was originally optimized for sheep embryo culture, Eagle’s MEM EAA and NEAA amino acids have become standard ingredients of SOF, even in bovine embryo culture (Table 5) [59]. SOF was subsequently optimized as a two-step culture media for bovine embryo culture: the SOF used for the first 72 h of culture is supplemented with taurine and EDTA (SOFC1), and taurine and EDTA-free SOF with increased glucose is used for subsequent culture (SOFC2) [56]. Another popular optimized SOF is SOF–bovine embryo 1 (SOF-BE1) with alanyl-glutamine, myo-inositol, and sodium citrate supplementation and the removal of glucose and glutamine [57,60]. The composition of selected modified SOF media is listed in Table 5.

## 7. Challenges and Future Perspectives

The ability to culture mammalian preimplantation embryos in vitro not only enables us to better understand the preimplantation development of mammalian embryos but also promotes the development of assisted reproductive technology. During the last several decades, remarkable progress in media formulation to simulate the in vivo growth environment of mammalian preimplantation embryos in vitro has been achieved. However, the current culture media are still not optimal. Compared to their in vivo environment, embryos in culture media are under stress as the culture environment, including the media, only partially represents the natural in vivo environment for their optimal development. Under suboptimal in vitro conditions, several abnormalities have been documented, such as slower mitosis than their in vivo counterpart, low developmental competence, an altered ultrastructure, abnormal gene expression during preimplantation development, etc. [23,61,62]. In recent years, as new understanding of the differences between in vivo- and in vitro-generated embryos at the molecular level emerges, prompted by new technologies, such as RNA-Seq [63], transcriptomic analysis [64,65], proteomics [66,67] and identification of molecules involved in embryo–maternal interaction [68], etc., the analysis and application of new knowledge can potentially improve the culture conditions and thus the quality of in vitro-generated embryos [69,70]. For example, the addition of purified osteopontin/secreted phosphoprotein-1 (SPP1) reduced polyspermy in porcine in vitro fertilization systems, as SPP1 was found to be rich in estrus oviduct by microarrays [71,72]. Therefore, improvements in culture conditions, including media optimization, are expected to continue as new methods and technology evolve to elucidate the dynamics of in vivo environments for preimplantation embryos as well as the assessment of the embryos’ quality.

## Figures and Tables

**Table 1:** Ringer’s solution and its selected derivatives (mM).

Ingredients	Ringer’s Solution	Ringer–Locke’s Solution (Mammalian)	Krebs–Ringer Bicarbonate	Tyrode’s Solution
NaCl	133.00	154.00	118.00	137.00
KCl	1.34	5.60	4.70	2.70
KH_2_PO_4_	--	--	1.20	--
NaH_2_PO_4_	--	--	--	0.417
MgSO_4_·7H_2_O	--	--	1.20	--
MgCl_2_	--	--	--	1.05
CaCl_2_	1.25	2.16	2.50	1.80
NaHCO_3_	2.76	3.6	24.90	12.00
Glucose	--	5.60	10.00	5.50

**Table 2:** Composition of media used for mouse embryo culture (mM) and their ability to support embryo development in vitro.

Ingredients	BMOC-2 *	M16	CZB	SOM **	KSOMaa	KSOMaa-Ala-Gln
NaCl	94.88	94.66	81.62	85	95.00	95
KCl	4.78	4.78	4.83	0.25	2.50	2.50
KH_2_PO_4_	1.19	1.19	1.18	0.35	0.35	0.35
MgSO_4_·7H_2_O	1.19	1.19	1.18	0.2	0.20	0.20
CaCl_2_	1.71	1.71	1.71	1.71	1.71	1.71
NaHCO_3_	25.00	25.00	25.00	25.00	25.00	4.00
Na lactate	25.00	23.28	31.30	10.00	10.00	10.00
Na pyruvate	0.25	0.33	0.27	0.2	0.20	0.20
Glucose	5.56	5.56	0.00	0.2	0.20	0.20
Glutamine	--	--	1.00	1.00	1.00	--
Ala-Gln	--	--	--	--	--	1.00
EDTA	--	--	0.11	0.01	0.01	0.01
Penicillin G (K salt) (mg/mL)		0.06	0.05		0.06	0.06
Streptomycin sulfate (mg/mL)		0.05	0.07		0.05	0.05
BSA (mg/mL)	1.00	4.00	5.00	1.00	1.00	1.00
MEM NEAA	--	--	--	--	0.5×	--
MEM EAA	--	--	--	--	0.5×	--
Mouse embryo culture in vitro	2-cell stage to blastocyst	2-cell stage to blastocyst	1-cell stage to blastocyst (random-bred)	1-cell stage to blastocyst (outbred and inbred)	1-cell stage to blastocyst (outbred and inbred)	1-cell stage to blastocyst (outbred and inbred)

* Brinster’s medium for ovum culture (BMOC)-2. ** Simplex optimized medium (SOM).

**Table 3:** Composition of media used for rat embryo culture (mM) and their ability to support embryo development in vitro.

Ingredients	R1ECM	mR1ECM	mR1ECM-BSA	mR1ECM-FBS	KSOM-R
NaCl	78.8	76.7	106.70	76.7	95
KCl	3.20	3.20	3.20	3.20	5
MgCl_2_·6H_2_O	0.50	0.50	0.50	0.50	--
MgSO_4_·7H_2_O	--	--	--	--	0.20
CaCl_2_	2.00	2.00	2.00	2.00	1.71
NaHCO_3_	25.00	25.00	25.00	25.00	25.00
Na Lactate	10.00	10.00	10.00	10.00	10
Na Pyruvate	0.50	0.50	0.50	0.50	0.20
Glucose	7.50	7.50	7.50	7.50	0.20
Glutamine	0.10	0.10	0.10	0.10	1.00
EDTA	--	--	--	--	0.01
PVA * (mg/mL)	1.00	1.00	--	--	--
Penicillin G K salt	--	--	--	--	0.06 mg/mL
BSA (mg/mL)	--	--	4.00	--	1.00
MEM NEAA 100×	--	10.00 mL	10.00 mL	10.00 mL	10.00 mL
MEM EAA 50×	--	20.00 mL	20.00 mL	20.00 mL	20.00 mL
Taurine	--	--	--	--	1
Glycine	--	--	--	--	1
Glutamate	--	--	--	--	1
Alanine					1
FBS	--	--	--	10%	--
Rat embryo culture in vitro	One-cell to blastocyst (outbred and inbred)	One-cell to blastocyst (outbred and inbred)	Beneficial for sperm penetration and pronuclear formation	Promote embryo development from 16-cell to blastocyst	One-cell to blastocyst (outbred and inbred)

* Polyvinyl alcohol.

**Table 4:** Composition of media used for porcine embryo culture (mM) and their ability to support embryo development in vitro.

Ingredients	mKRB	NCSU-23	PZM-3	PZM-4
NaCl	94.00	108.73	108.00	108.00
KCl	4.78	4.78	10.00	10.00
KH_2_PO_4_	1.20	1.19	0.35	0.35
MgSO_4_·7H_2_O	1.20	1.19	0.40	0.40
CaCl_2_	2.50	1.70	--	--
NaHCO_3_	25.07	25.07	25.07	25.07
Na Lactate	29.2	--	--	--
Ca-(lactate)_2_·5H_2_O	--	--	2.00	2.00
Na Pyruvate	0.25	--	0.20	0.20
Glucose	5.56	5.55	--	--
Glutamine	--	1.00	1.00	1.00
Taurine	--	7.00	--	--
Hypotaurine	4	5.00	5.00	5.00
MEM NEAA 50× (mL/L)	--	--	20.00	20.00
MEM EAA 100× (mL/L)	--	--	10.00	10.00
BSA (mg/mL)	4	--	3.00	--
PVA (mg/mL)	--	--	--	3.00
Porcine embryo culture in vitro	4-cell stage to blastocyst	1-cell stage to blastocyst	1-cell stage to blastocyst	1-cell stage to blastocyst

**Table 5:** Composition of major modified SOF media (mM) for bovine embryo culture and their ability to support embryo development in vitro.

Ingredients	SOF	SOFaa	SOFC1	SOFC2	SOF-BE1
NaCl	107.70	107.70	99.70	99.70	107.70
KCl	7.16	7.16	7.16	7.16	7.16
KH_2_PO_4_	1.19	1.19	1.19	1.19	1.19
MgCl_2_·6H_2_O	0.49	0.49	0.49	0.49	0.49
CaCl_2_·H_2_O	1.71	1.71	1.71	1.71	1.17
NaHCO_3_	25.07	25.07	25.07	25.07	25.07
Na Lactate	3.30	3.30	3.30	3.30	5.30
Na Pyruvate	0.33	0.33	0.33	0.33	0.40
Sodium citrate	--	--	--	--	0.50
Glucose	1.50	1.50	1.50	3.00	--
Glutamine	--	1.00	1.00	1.00	--
Alanyl-Glutamine	--	--	--	--	1.00
Myo-inositol	--	--	--	--	2.77
EDTA	--	--	0.1	--	--
Taurine	--	--	0.1	--	--
Penicillin Na salt (unit/mL)	100	100	--	--	--
Streptomycin (µg/mL)	50	50	--	--	--
Gentamicin sulfate (µg/mL)	--	--	--	--	25.00
MEM NEAA 50× (mL/L)	20	20	20	20	20
MEM EAA 100× (mL/L)	10	10	10	10	10
BSA (mg/mL)	32	32	8	8	4
Bovine embryo culture in vitro	8-cell stage to blastocyst	1-cell stage to blastocyst	1-cell stage to blastocyst	1-cell stage to blastocyst	1-cell stage to blastocyst

## Data Availability

No new data were created or analyzed in this study. Data sharing is not applicable to this article.
